# Molecular mechanism of *nur77* gene expression and downstream target genes in the early stage of forskolin-induced differentiation in PC12 cells

**DOI:** 10.1038/s41598-020-62968-y

**Published:** 2020-04-14

**Authors:** Hiroki Maruoka, Ryosuke Yamazoe, Ryota Takahashi, Keisuke Yatsuo, Daiki Ido, Yuki Fuchigami, Fumiya Hoshikawa, Koji Shimoke

**Affiliations:** Laboratory of Neurobiology, Department of Life Science and Biotechnology, Faculty of Chemistry, Materials and Bioengineering, Kansai University, 3-3-35, Yamate-cho, Suita, Osaka, 564-8680 Japan

**Keywords:** Cellular neuroscience, Molecular neuroscience

## Abstract

Forskolin promotes neuronal differentiation of PC12 cells via the PKA-CREB-dependent signaling pathway. Activation of PKA by forskolin phosphorylates CREB, which then binds to CRE sites in numerous gene promoters. However, it is unclear which gene contains the CRE sites responsible for forskolin-induced neuronal differentiation. In this study, we investigated how an immediate early gene, *nur77*, which has CRE sites in the promoter region, contributes to the early stage of differentiation of forskolin-treated PC12 cells. After treatment with forskolin, expression of Nur77 was upregulated within 1 hr. In addition, knockdown of *nur77* inhibited neurite outgrowth induced by forskolin. We also revealed that the specific four CRE sites near the transcriptional start site (TSS) of *nur77* were strongly associated with phosphorylated CREB within 1 hr after treatment with forskolin. To analyze the roles of these four sites, reporter assays using the *nur77* promoter region were performed. The results showed that *nur77* expression was mediated through three of the CRE sites, −242, −222, and −78, and that −78, the nearest of the three to the TSS of *nur77*, was particularly important. An analysis of neuronal markers controlled by Nur77 after A-CREB-Nur77-Synapsin1 signaling pathway plays a pivotal role in differentiation of forskolin-induced PC12 cells.

## Introduction

Low molecular weight natural products may be useful therapeutic agents for neuronal injury^1–6^, and some of these compounds possess neurotrophic and neuroprotective properties^7^. Natural products also enhance neurite outgrowth activity of nerve growth factors in experimental models^8^, but the detailed molecular mechanisms underlying the neurotrophic and neuroprotective effects of natural products have not been clearly defined.

One such natural product, forskolin, is a cell-permeable diterpenoid extracted from the plant Coleus forskohlii. Forskolin also has blood-brain barrier (BBB) permeability^9^. Therefore, forskolin is a potential therapeutic agent for nerve injury, and several studies have shown that forskolin increases the differentiation and survival of dopaminergic neurons *in vitro*^10–14^. Forskolin also induces expression of tyrosine hydroxylase in human fetal brain cortex^15^. In addition, forskolin increases the intracellular cAMP level by stimulation of adenylate cyclase, and cAMP-dependent signaling pathways play important roles in neuronal differentiation and neuroplasticity^16,17^. Increased cAMP in cells promotes axonal regeneration and neurite outgrowth^18–20^, and it is well-known that cAMP promotes neurite outgrowth by binding to and activating protein kinase A (PKA)^21–23^.

Activated PKA phosphorylates cAMP response element-binding protein (CREB), which then binds to CRE (cAMP response element) sites in various gene promotor regions^24–26^. Some CREB target genes have been identified as immediate early genes (IEGs) that are induced in a CRE-dependent manner. Expression of IEGs can be induced within 1 hr in response to stimuli such as neurotrophin/Trk- or cAMP/PKA-dependent signaling pathways^27–32^. Recent studies in our laboratory have shown that one IEG, *nur77*, is upregulated by dibutyryl-cAMP (db-cAMP) via the cAMP/PKA-dependent signaling pathway in model neuronal PC12 cells. In addition, we found that expression of Nur77 is essential for the early phase of neurite extension in PC12 cells^20,33–35^.

Nur77 is an orphan nuclear receptor that is also referred to as NGFI-B, TR3 and Nr4a1. Nur77 is a member of the Nur77 family, which also contains the orphan nuclear transcription factors Nurr-1 and Nor-1. Nur77 was originally identified as a protein that is rapidly induced by nerve growth factors in PC12 cells^36,37^ and by serum in fibroblasts^38^. Recent studies have shown that Nur77 expression is regulated by CRE-binding proteins. The *nur77* gene contains four CRE sites (TGCGTCA; previously defined as the AP1 (activator protein 1) element) upstream of the transcription start site (TSS)^26,39,40^. These four CRE sites have been suggested to bind CREB protein^25,26,37,41^, and binding of transcription factors at the four CRE sites in the *nur77* promoter may play an important role in the early stage of forskolin-induced neuronal differentiation. However, the detailed mechanisms of *nur77* transcription in the early phase of neurite extension are largely unknown, and the relevance of *nur77* and its transcription factors in neural differentiation is not understood.

In this study, we investigated the mechanism underlying regulation of *nur77* transcription during the early phase of neurite extension induced by forskolin in PC12 cells. We found that the four CRE sites upstream of the TSS of *nur77* are associated with phosphorylated CREB (P-CREB) within 1 hr after treatment with forskolin. We also found that the −242, −222 and −78 CRE sites, and especially −78, play particularly important roles. To identify the critical molecules regulated by Nur77 during forskolin-induced neurite extension, Nur77 regulation of proteins that serve as neuronal differentiation markers was analyzed. The findings showed that Nur77 regulated one such protein, Synapsin 1, but did not influence β-tubulin III or NeuroD, although it was reported that β-tubulin III or NeuroD was expressed under the Nur77 regulation. These results suggest that the PKA-CREB-Nur77-Synapsin1 signaling pathway is essential for forskolin-induced differentiation of PC12 cells, including neurite extension.

## Results

### Nur77 is involved in neurite outgrowth induced by forskolin in PC12 cells

**Figure 1:**
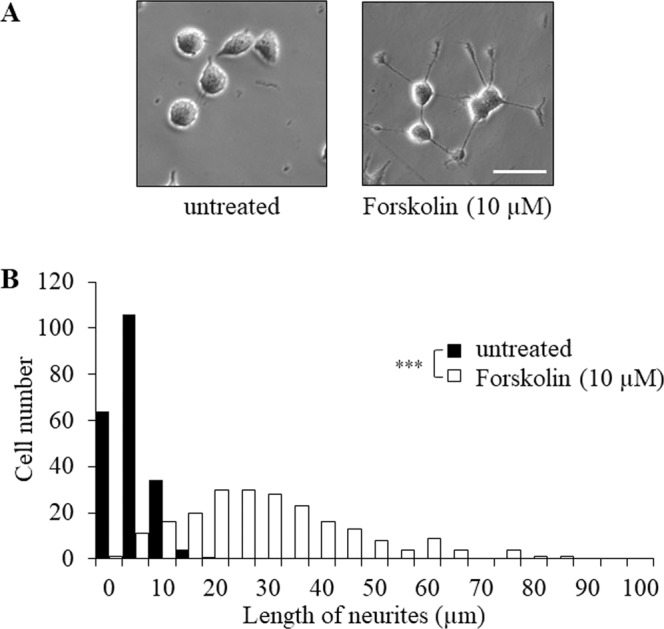
Nur77 is involved in neurite outgrowth induced by forskolin in PC12 cells. (**A)** Photomicrographs of PC12 cells cultured for 24 hr without or with 10 µM forskolin. Scale bar: 50 μm. (**B**) Histograms of neurite lengths in PC12 cells cultured for 24 hr without (closed bars: untreated) or with 10 μM forskolin (open bars: forskolin-treated). For analysis of neurite outgrowth, cells (more than 200 /well) were randomly photographed using a KEYENCE microscope. The lengths of neurite were measured using BZ-H1C software. ***P < 0.001 (untreated vs. forskolin-treated cells by Kolmogorov-Smirnov test).

To confirm if forskolin has a role in neurite outgrowth, the lengths of neurites were measured after treatment with forskolin in PC12 cells. Neurite lengths of PC12 cells treated with 10 µM forskolin for 24 hr were significantly greater than those from untreated cells (Fig. 1A,B and see Supplementary Table S1 on line) as reported previously^42,43^.

**Figure 2:**
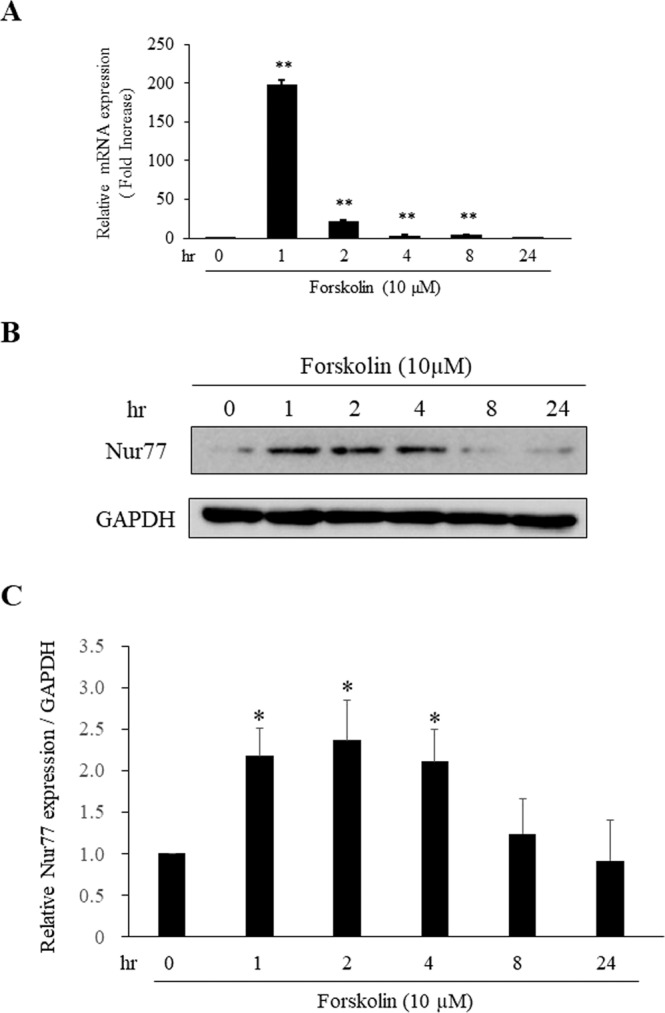
The expression of *nur77* gene and Nur77 are induced at 0–4 hr after 10 µM forskolin treatment in PC12 cells. (**A**) PC12 cells were treated with 10 μM forskolin. *nur77* and *gapdh* mRNA were detected by qPCR as described in Methods section. *nur77* mRNA levels were normalized against *gapdh* mRNA levels and against the initial time point (0 hr). **P < 0.01. (**B**) For immunoblot analysis of forskolin-induced Nur77 in PC12 cells, cells were treated with 10 μM forskolin for the indicated times in DMEM supplemented with 1% (v/v) FBS. (**C**) Quantification of B, comparing the protein levels of Nur77 protein in the indicated times. The amount of Nur77 protein was quantified and normalized to that of GAPDH. *P < 0.05 compared with 0 hr.

Previous studies have shown that expression of *nur77* is significantly increased by db-cAMP in PC12 cells, and that Nur77 is essential for early stage of differentiation in neurons and Schwann cells^20,34,35,44^. To investigate whether the increased expression of Nur77 is mainly responsible for neurite outgrowth that occurs from 0 to 24 hr after 10 µM forskolin treatment, expression of *nur77* gene and Nur77 induced by forskolin was examined using qPCR and immunoblotting analysis (Fig. 2A–C). The peak expression of *nur77* gene and Nur77 after forskolin treatment was reached at 1–4 hr. These data suggest that expression of *nur77* gene and Nur77 are induced at 0–4 hr after 10 µM forskolin treatment.

### Binding of CREB with CRE sites near the TSS of *nur77* is responsible for forskolin-induced Nur77 activation and neurite outgrowth

**Figure 3:**
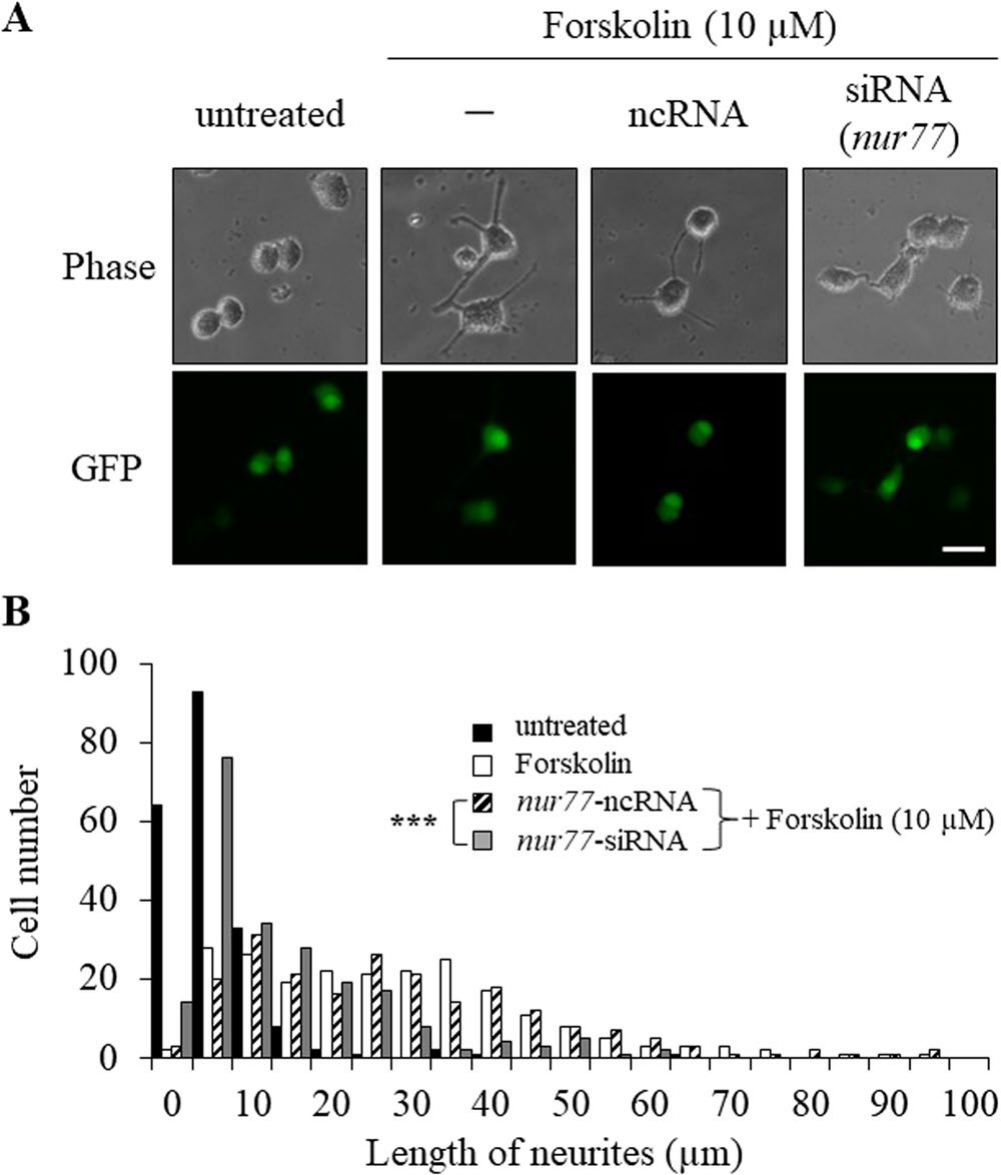
Nur77 is involved in forskolin-induced neurite outgrowth in PC12 cells. (**A**) After co-transfection with siRNAs against *nur77* mRNA or ncRNA containing a GFP-expression plasmid for 48 hr, cells were treated with or without (untreated) 10 μM forskolin in DMEM supplemented with 1% (v/v) FBS for 24 hr. Phase contrast (Phase) and GFP-expression (GFP) images were detected and photographed. Scale bar: 50 μm. (**B**) Histograms of neurite lengths of forskolin-treated PC12 cells (open bars) pretreated with siRNA against *nur77* mRNA (gray bars) and ncRNA control (hatched bars). PC12 cells pretreated with siRNA or ncRNA in the presence of a GFP-expression plasmid for 48 hr were then treated with 10 μM forskolin for a further 24 hr. PC12 cells were cultured without (closed bars: untreated) or with 10 μM forskolin (open, hatched and gray bars). For analysis of neurite outgrowth, cells (more than 200cells /well) were randomly photographed using a KEYENCE microscope. The lengths of neurite were measured using BZ-H1C software. ***P < 0.001 (*nur77* siRNA vs. ncRNA).

To analyze the details of the function of Nur77 in the early period of forskolin treatment, knockdown experiments were performed using siRNA against *nur77* mRNA. First, it was confirmed that knockdown of *nur77* mRNA inhibited neurite outgrowth after treatment with forskolin. Neurite length in cells treated with siRNA against *nur77* mRNA in the presence of forskolin was significantly lower than that in cells treated with negative universal control siRNA (Fig. 3A,B and see Supplementary Table S2 on line). These results indicate that upregulation of Nur77 is required for differentiation of PC12 cells by 10 µM forskolin.

CRE sites near the TSS are known to be heavily involved in regulation of transcription. In PC12 cells, we have shown that the CRE site-containing *nur77* promoter region near the TSS is associated with acetylated Lys14 of histone H3 after treatment with db-cAMP or HDAC (Histone Deacetylase) inhibitors TSA and K350; and that expression of Nur77 induced by db-cAMP is regulated by the PKA-CREB pathway^20,34,35^. Based on these findings, it is likely that CRE sites upstream of the TSS of *nur77* and CREB are important for *nur77* expression.

**Figure 4:**
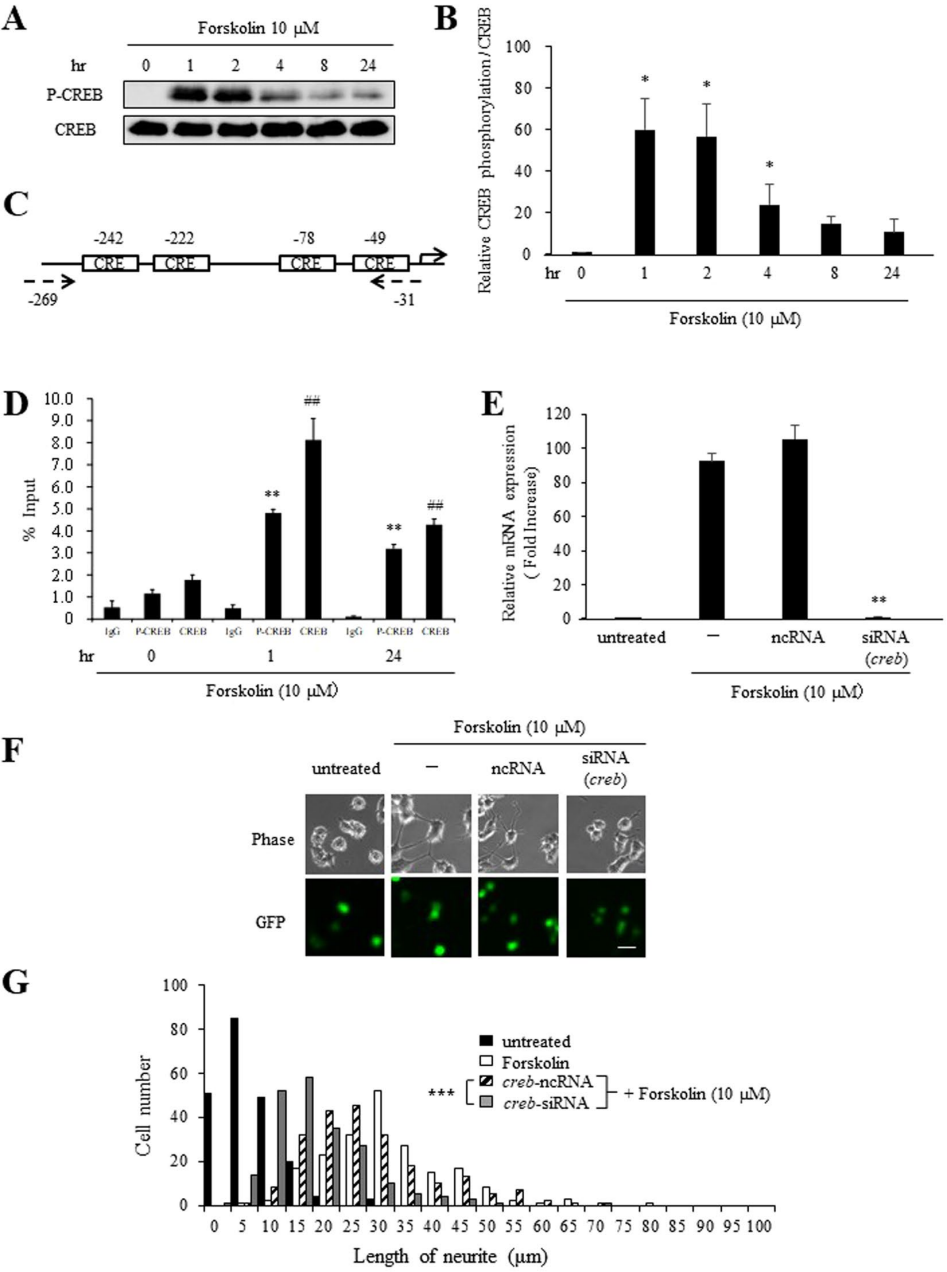
Binding of P-CREB to the CRE sites of the *nur77* promoter plays an important role in transcriptional activation of *nur77*. (**A**) Immunoblot analysis of forskolin-induced P-CREB in PC12 cells. Cells were treated with 10 μM forskolin for the indicated times. (**B**) Quantification of A, comparing the phosphorylation level of P-CREB protein in the indicated times. The amount of P-CREB protein was quantified and normalized to that of CREB. *P < 0.05 compared to 0 hr. (**C**) Schematic diagram of the *nur77* gene promoter region. There are four CRE sites at −242, −222, −78 and −49 bp upstream of the *nur77* gene. The primers used in ChIP assays were designed to detect the region from −31 and −269 bp from the TSS. (**D**) Cells were treated with 10 μM forskolin for the indicated times. Cells were then subjected to ChIP assays with an antibody against P-CREB and CREB as described in Methods. The *nur7*7 gene promoter region was detected by qPCR. **P < 0.01 compared with 0 hr (P-CREB), ^##^P < 0.01 compared to 0 hr (CREB). € Cells transfected with *creb* siRNA or ncRNA were treated with or without 10 μM forskolin for 1 hr. *creb* and *gapdh* mRNA were detected by qPCR. *creb* mRNA were normalized against *gapdh* mRNA. **P < 0.01 compared with ncRNA. (**F**) After co-transfection with *creb* siRNA or ncRNA containing a GFP-expression plasmid for 48 hr, cells were treated with or without 10 μM forskolin for 24 hr. Phase contrast (Phase) and GFP-expression (GFP) images were detected and photographed. Scale bar: 50 μm. (**G**) Histograms of neurite lengths of forskolin-treated cells (open bars) pretreated with *creb* siRNA (gray bars) and ncRNA (hatched bars). Cells pretreated with siRNA or ncRNA in the presence of a GFP-expression plasmid for 48 hr were then treated with forskolin for a further 24 hr. Cells were cultured without (closed bars: untreated) or with 10 μM forskolin (open, hatched and gray bars). For analysis of neurite outgrowth, cells (more than 200 cells /well) were randomly photographed. ***P < 0.001 (*creb* siRNA vs. ncRNA).

To explore the role of these CREs sites and CREB in the early stage of forskolin-induced differentiation of PC12 cells, immunoblotting analysis using anti-phosphorylated CREB antibody was first performed. P-CREB was first observed 1 hr after treatment with 10 µM forskolin, similar to expression of Nur77, and phosphorylation reached a peak at 1–4 hr and then declined to the basal level after 4 hr (Fig. 4A,B). To examine the contribution of P-CREB to 10 µM forskolin-induced *nur77* expression mediated by CRE sites, a ChIP assay using anti-P-CREB and CREB antibody was carried out. This assay revealed that the *nur77* promoter region containing the CRE sites (Fig. 4C) was particularly associated with P-CREB within 1 hr after treatment with 10 µM forskolin (Fig. 4D). The ChIP assay also showed that the *nur7*7 promoter region was associated with CREB with or without 10 µM forskolin treatment (Fig. 4D). Taken together, these results suggest that binding of P-CREB to the CRE sites of the *nur77* promoter plays a particularly important role in mediating transcriptional activation of *nur77*.

To analyze the details of the role of CREB in the early period of 10 µM forskolin induced *nur77* gene expression and neurite outgrowth, knockdown experiments were performed using siRNA against *creb* mRNA. First, we analyzed the relationship between *nur77* gene expression and CREB. After treatment with *creb* siRNA for 24 hr in the presence or absence of forskolin, *nur77* gene reduced strongly compared with cells treated with a negative control siRNA (Fig. 4E). Next, we confirmed that neurite length in cells treated with siRNA against *creb* mRNA in the presence of forskolin was significantly lower than that in cells treated with negative control siRNA (Fig. 4F,G and see Supplementary Table S3 on line). These results suggest that CREB has an important role for *nur77* gene expression in differentiation of 10 µM forskolin-treated PC12 cells.

### CRE sites at −242, −222 and −78 play a pivotal role in forskolin-induced nur77 expression

**Figure 5:**
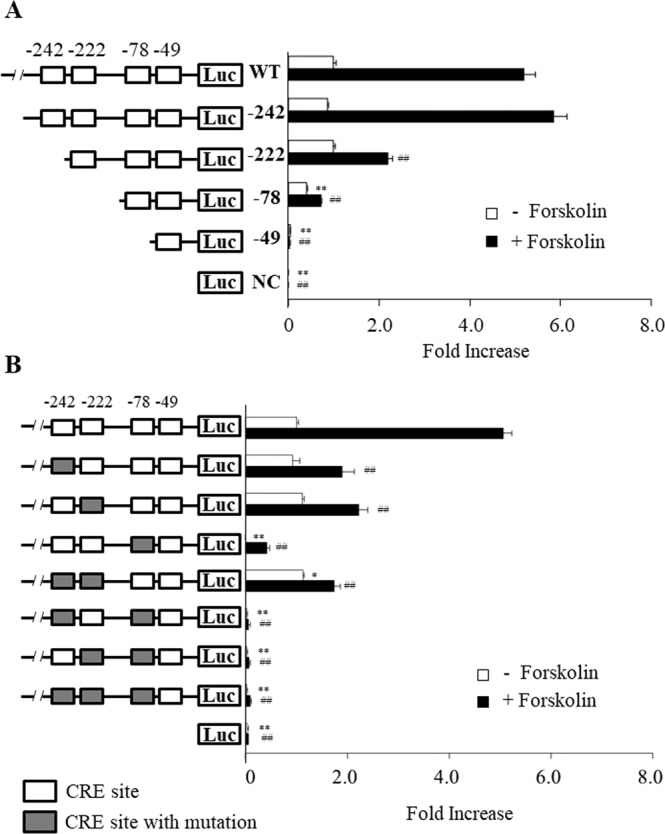
The promoter region between −242 and −78, and particularly at −78 have a pivotal role of *nur77* gene expression in PC12 cells. (**A**) Deletion analysis of Nur77 gene promoter region. Constructs used in this experiment are schematically drawn and were prepared as described in Methods. Cultured PC12 cells were transiently transfected with reporter plasmids and phRL-TK plasmid. Cells were treated with or without 10 μM forskolin. Each CRE site is drawn as a square in the promoter region. **P < 0.01 compared with WT (−Forskolin), ^##^P < 0.01 compared to WT (+Forskolin). (**B**) Effects of mutations in −242, −222 and −78 CRE sites within the promoter region of the rat nur77 gene. The mutant promoter plasmids positioned at −242, −222 and −78 CRE sites are shown schematically. Mutations in CRE sites are shown as gray boxes. Cultured PC12 cells were transiently transfected with reporter plasmid and phRL-TK plasmid. Cells were treated with or without 10 μM forskolin. **P < 0.01 and *P < 0.05 compared to WT (−Forskolin), ^##^P < 0.01 compared with WT (+Forskolin).

The CRE sites upstream of the TSS of *nur77* are located at positions −242, −222, −78 and −49 (Fig. 4B). To identify which of are important in Nur77 transcriptional activity induced by 10 µM forskolin, a reporter assay was carried out. To analyze the role of the *nur77* promoter in forskolin-induced neurite outgrowth, luciferase reporter plasmids were constructed with the four CRE sites deleted one by one (Fig. 5A). Strong Nur77-related luciferase activities were observed with the WT and −242 plasmid with or without treatment with forskolin. In contrast, the −222, −78 and −49 plasmids had marked reduction of luciferase activity with treatment of forskolin, and −78 and −49 had reduced luciferase activity without forskolin (Fig. 5A). With plasmid −49, luciferase activity was completely abolished. Therefore, the regulatory elements for *nur77* expression appear to be present in the promoter region between −242 and −78.

We next examined which of these three CRE sites in the promoter play significant roles in *nur77* expression. As shown in Fig. 5B, luciferase activity was measured using promoter assay plasmids in which mutations were interpolated in one or two or all of the three distal CRE sites. Plasmids with a point mutation at any one of three CRE sites −242, −222 or −78 caused a reduction in luciferase activity under forskolin-treated condition, but particularly marked reduction occurred with the −78 mutant. In contrast, under untreated conditions, the luciferase activity was markedly reduced only with the −78 mutant. With mutants at two or three CRE sites, luciferase activity was completely abolished when a mutation at the −78 CRE site was included (Fig. 5B). These results suggest that the regulatory elements of *nur77* expression are present in the promoter region between −242 and − 78, and particularly at −78.

### The Nur77-Synapsin signaling pathway is required for forskolin-dependent neuronal differentiation

**Figure 6:**
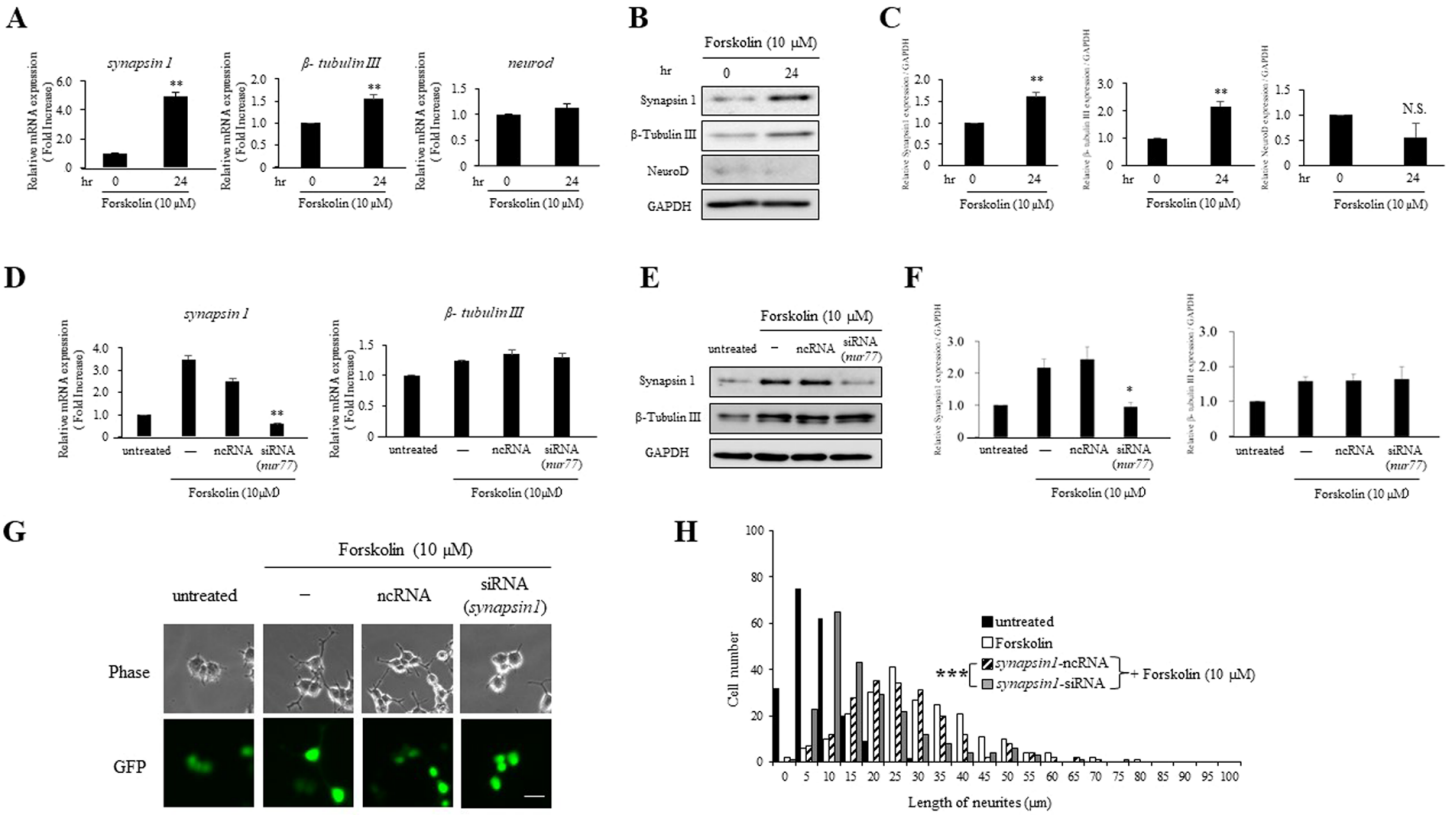
The Nur77-Synapsin1 signaling pathway has an important role in differentiation of forskolin-treated PC12 cells. (**A**) Cells were treated with 10 μM forskolin. *synapsin1, β-tubulin III, neurod* and *gapdh* mRNA were detected by qPCR. mRNAs were normalized against *gapdh* mRNA. **P < 0.01 compared with 0 hr. (**B**) Immunoblot analysis of forskolin-induced Synapsin1, β-tubulin III and NeuroD. Cells were treated with 10 μM forskolin for 24 hr. (**C**) Quantification of B, comparing the protein levels of Synapsin1, β-tubulin III, NeuroD and GAPDH protein in the indicated times. The amount of protein was quantified and normalized to that of GAPDH. **P < 0.01 compared with 0 hr., N.S. denotes not significant for statistical analysis. (**D**) Cells transfected with *nur77* siRNA were incubated in the presence or absence of 10 μM forskolin for 24 hr. *Synapsin1, β-tubulin III* and *gapdh* mRNA were detected by qPCR. mRNAs were normalized against *gapdh* mRNA. **P < 0.01 compared with untreated. (**E**) Cells transfected with *nur77* siRNA were incubated in 10 μM forskolin for 24 hr. Synapsin1, β-tubulin III and GAPDH were detected by immunoblot analysis. These bands were representatives of three independent experiments. (**F**) Quantification analysis of the protein levels of Synapsin1 and β-Tubulin III was performed (n = 3). The amount of these protein was quantified and normalized to that of GAPDH. *P < 0.05 compared with untreated. (**G**) After co-transfection with *synapsin1* siRNA or ncRNA containing a GFP-expression plasmid for 48 hr, cells were treated with 10 μM forskolin for 24 hr. Phase contrast (Phase) and GFP-expression (GFP) images were detected and photographed. Scale bar: 50 μm. (**H**) Histograms of neurite lengths of forskolin-treated PC12 cells (open bars) pretreated with *synapsin1* siRNA (gray bars) and ncRNA (hatched bars). Cells pretreated with siRNA or ncRNA in the presence of a GFP-expression plasmid for 48 hr were then treated with forskolin for a further 24 hr. Cells were cultured without (closed bars: untreated) or with 10 μM forskolin (open, hatched and gray bars). For analysis of neurite outgrowth, cells (more than 200 cells /well) were randomly photographed. ***P < 0.001 (*synapsin1* siRNA vs. ncRNA).

The effect of Nur77 on neuron-specific markers was analyzed in neurite outgrowth induced by forskolin. We first examined if the neuron-specific marker, Synapsin1 as a synaptic marker^45^, β-tubulin III as a neuronal marker of newly generated neurons^46–48^ or NeuroD as a neuronal marker for the early phase of the neuronal lineage^48^, was upregulated with treatment of 10 µM forskolin for 24 hr. qPCR and immunoblotting detected Synapsin1 and β-tubulin III, but not NeuroD (Fig. 6A–C). After treatment with *nur77* siRNA for 24 hr in the presence or absence of forskolin, immunoblotting showed strong reduction of Synapsin1 compared with cells treated with a negative control siRNA. In contrast, the β-tubulin III level was not altered by treatment with *nur77* siRNA (Fig. 6D–F).

To analyze the details of the role of Synapsin1 in the early period of 10 µM forskolin treatment, knockdown experiments were performed using siRNA against *synapsin1* mRNA. We examined that neurite length in cells treated with siRNA against *synapsin1* mRNA in the presence of forskolin was significantly lower than that in cells treated with negative control siRNA (Fig. G, H and see Supplementary Table S4 on line).

These results suggest that the Nur77-Synapsin1 signaling pathway, but not β-tubulin III or NeuroD, has an important role in differentiation of forskolin-treated PC12 cells.

## Discussion

Therapeutic agents for nerve injury must have two main characteristics of passage through the BBB and induction of neuronal differentiation. Forskolin has these properties and is also a natural product, which may reduce side effects. Therefore, this compound may be a useful drug for nerve injury. Determination of the intracellular mechanism of differentiation induced by forskolin is needed as part of the risk assessment for this potential drug. In this study, we showed that Nur77 has an important role in forskolin-induced differentiation, and we found that binding of P-CREB to the *nur77* promoter region and expression of Nur77 occurred within 1 hr of forskolin treatment. In particular, we suggest that binding of P-CREB at the −242, −222 and −78 CRE sites among the four CRE sites in the *nur77* promoter region near the TSS is essential for forskolin-induced *nur77* gene activity.

The CRE sites near the TSS of *nur77* have previously been shown to be important in *nur77* transcription, but this study is the first to show that binding of CREB at specific CRE sites has a pivotal role in the early stage of forskolin-induced neurite outgrowth. Three of the four CRE sites in the *nur77* promoter between −274 and −69 from the TSS have previously been shown to have important roles in response to extracellular stimuli^25,40,41^. Similarly, our data showed that the three CRE sites at −242, −222 and −78 play important roles in forskolin-induced differentiation in PC12 cells (Fig. 5B). These results suggest that these three CRE sites have especially pivotal roles in *nur77* expression. To understand the details of the mechanism of Nur77 expression, it will be important to know which transcription factors bind to these three CRE sites. It has recently been reported that binding of CREB to these CRE sites is enhanced by 8 Br-cAMP, PMA, EGF and TNF treatment^25,41^.

CREB plays an important role in differentiation and is also an essential mediator of Ca^2+^-activated Nur77 expression in PC12 cells and binds to the endogenous *nur77* promoter region *in vivo*. Moreover, CRE sites in the *nur77* promoter contain the central CpG that is critical for CREB binding^25,26,49^. Our previous data showed that the PKA-CREB-Nur77 pathway is important in neurite outgrowth in the db-cAMP-induced mechanism in PC12 cells^20^. Binding of Jun family proteins to CRE sites in the *nur77* promoter is also enhanced by 8Br-cAMP^25^. One such protein, JunD, is an atypical member of the AP1 family that can act as both a transcriptional activator and repressor, and is also activated by an elevated level of intracellular cAMP^50^. c-jun, another Jun family member, is a master regulator of neurite outgrowth and axonal regeneration in nerve injury^51,52^. However, there is no direct evidence that binding of CREB or Jun proteins during the upregulation of Nur77 expression is involved in the differentiation, and it is uncertain of there is involvement of gene regulators other than the CREB or Jun family. Further detailed studies are required in this area.

IEGs other than Nur77 are also involved in differentiation. For example, Egr1 is an IEG that induces neuronal differentiation and causes neurite outgrowth in N2 neuroblastoma cells^53^. The induction of Egr1 via the MAPK-ERK pathway has been linked to neuronal differentiation and plasticity. Two other *nur77* family genes, *nurr1* and *nor-1*, have also been shown to act as IEGs, and are correlated with neuronal differentiation. *nurr1* is required for the development and survival of dopaminergic neurons^54,55^, and upregulation of *nurr1* in neuronal precursor cells promotes undifferentiated cells into mature and functional dopaminergic neurons^56^. Consistent with this, *nurr1*-deficient mice exhibit abnormal development of midbrain dopaminergic neurons^54^. *nor-1* also plays a critical role in neuronal survival and axonal guidance in the developing murine hippocampus^57^, and is also involved in regulating neuronal differentiation during extension of processes in cultured fetal rat brain cells^58^. These findings indicate that Nur77 family members are involved in several aspects of neuronal differentiation in the developing nervous system.

Similarly to *nur77*, the *nurr1* and *nor-1* genes also have DNA motifs near the TSS that bind with transcriptional factors, such as CREB, with the transcriptional activity that is increased by phosphorylated PKA. The *nurr1* promoter contains a TGACG motif near the TSS, and the *nor-1* promoter has three CRE sites (TGACGTAG, TGGCGTCA, and TGACGTCT) near the TSS^59^. This suggests that the same transcription factor, CREB, binds to CRE sites in these Nur77 family members and may participate in neuronal differentiation. Therefore, the Nur77 family may have a common molecular mechanism in neural differentiation. However, Nur77 family members also show some differences in transcriptional activities, despite having highly homologous amino acid sequences^41,60^. These differences are thought to be due to the involvement of DNA binding domains other than the CRE sites. For example, *nur77* and *nor-1* contain binding sites for MEF2 (MEF2 response elements, MRE) near the TSS and the CRE sites in their promoter regions^26,61^. Early work suggested that MEF2 modulates CREB-dependent Nur77 expression by acting as a repressor in quiescent cells, while CREB is necessary for Ca^2+^-activated Nur77 expression^26^. Therefore, to define the relationship between the mechanism of *nur77* gene expression in neuronal differentiation and the DNA motifs near the TSS, a detailed analysis of the CRE sites and the relationships between other DNA binding motifs around these sites and the relevant transcription factors is required.

**Figure 7:**
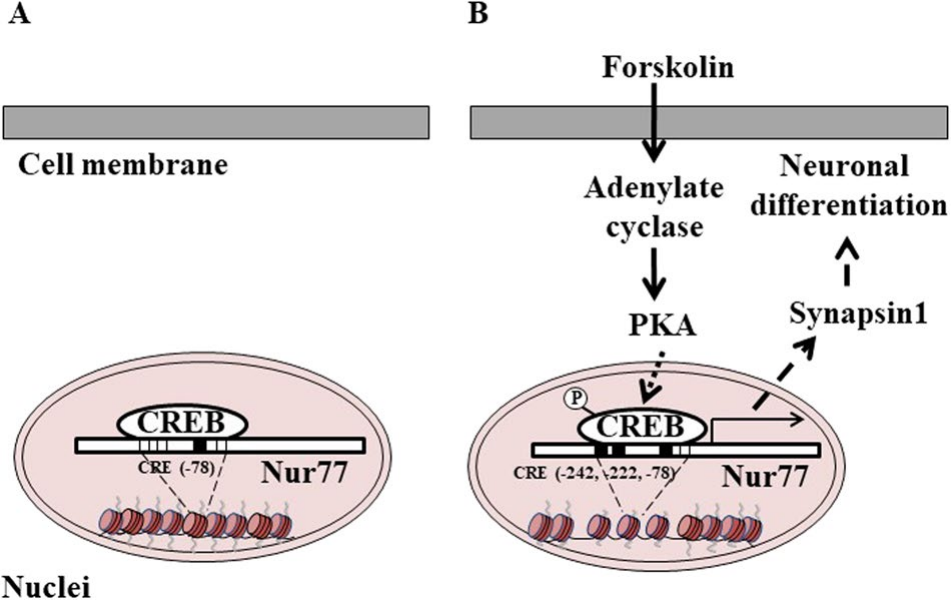
The *nur77* gene expression-dependent molecular pathway to forskolin-induced neuronal outgrowth. (**A**) In the forskolin-untreated (basal) condition, CREB binds to the −78 CRE site (black closed box in the nucleus) in the *nur77* promoter region, causing expression of Nur77. (**B**) After stimulation with forskolin, the PKA-CREB pathway is activated through adenylate cyclase production. Then, CREB is phosphorylated (P-CREB) and the P-CREB binds to the three CRE sites in the *nur77* gene upstream of the TSS. The binding sites (−242, −222 and −78) are shown as black closed boxes in the nucleus. Binding of phosphorylated CREB to the *nur77* gene promoter region strongly induces Nur77 expression, after which Nur77 upregulates expression of Synapsin1 to promote differentiation of PC12 cells.

In this study, we also found that the Nur77 upregulated Synapsin1, but not β-tubulin III or NeuroD, as a key step during forskolin-induced neurite outgrowth in PC12 cells. This finding provides direct evidence that Nur77 is involved in the differentiation of PC12 cells. Synapsin is an abundant pre-synaptic phosphoprotein associated with the cytoplasmic side of synaptic vesicles that promotes actin polymerization and triggers cell differentiation^62–65^. Synapsin1 is a useful indicator of PC12 cell differentiation^66^, and post-translational modifications of Synapsin1 by PKA contribute to the regulation of neurotransmitter release^67^. These results lead us to speculate on the following model (Fig. 7). Under basal conditions, CREB binds to the promoter region of *nur77* via the −78 CRE site (Fig. 7A). If forskolin activates the PKA-CREB pathway, CREB is phosphorylated and binds to CRE sites upstream of the TSS in *nur77*. Subsequently, Nur77 expression is induced via binding of P-CREB at the −244, −222, and −78 CRE sites of the *nur77* promoter. Induced Nur77 may then upregulate the expression of Synapsin1 and cause differentiation of neurons (Fig. 7B). However, it is unclear if Synapsin1 is directly upregulated by Nur77 or regulated by a transcription factor controlled by Nur77. An answer to this questions requires further analysis of the molecular mechanism of the Nur77-Synapsin1 signaling pathway in forskolin-induced neuronal differentiation.

In this study, we also revealed that NeuroD was not involved in forskolin-induced neurite outgrowth of PC12 cells. However, previous study demonstrated that Bcl-2 regulates neurite outgrowth through the Bmp4 - Tbx3 - NeuroD1 cascade in H19-7 cells^68^. In addition, it has been known that Nur77 is localized to the mitochondria, inducing the release of Cytochrome C and Caspase 3 through interaction with Bcl-2. This molecular interaction causes apoptosis in following apoptosis stimulation^69,70^. These findings suggest that the function or the expression of NeuroD may be regulated by Nur77 in neurite outgrowth that does not involve in the PKA-CREB pathway. Furthermore, we demonstrated that β-tubulin III was not regulated by Nur77 in forskolin-induced neurite outgrowth of PC12 cells. On the other hand, Damodaran *et al*. have reported that phosphorylation of CREB by PKA and a member of the CaMK family, CaMKII play a crucial role in the altered axonal transport by modulating the transcription of β-tubulin gene^71^. In addition, it has also been shown that CaMKI pathway contributes to cAMP-induced Nur77 expression in MA-10 Leydig cells^72^. These findings suggest that Nur77 may regulate the expression of tubulin in neurite outgrowth induced by activators of the PKA-CREB pathway. There are numerous genes that are regulated by Nur77 in neural differentiation, and their regulatory mechanisms are still unclear. Therefore, we need more detailed analyses about the downstream molecules of Nur77 in future.

The findings in this study reveal that the PKA-CREB-Nur77 pathway is essential for forskolin-induced differentiation of PC12 cells. We also showed that expression of *nur77* is initiated by binding of CREB to the CRE sites −242, −222 and −78, and especially −78, in the *nur77* promoter region near the TSS. We also found that Nur77 upregulated Synapsin1, a neuronal differentiation marker. Further detailed studies are needed to define the molecular mechanisms of Nur77-mediated differentiation induced by forskolin.

## Methods

### Cell culture

PC12 cells were maintained in Dulbecco’s modified Eagle’s medium (DMEM) supplemented with 5% (v/v) fetal bovine serum (FBS) (Sigma, MO, USA), 5% (v/v) horse serum, 10 mM HEPES (pH7.0) and 0.1% (v/v) penicillin-streptomycin (Gibco BRL, Carlsbad, CA).

### Measurement of neurite outgrowth

Cells were seeded in 24-well plates at 1.5 × 10^4^ cells/cm^2^. After 16 hr, the medium was replaced with DMEM in the presence or absence of 10 µM forskolin (Sigma, MO, USA) supplemented with 1% (v/v) FBS. The cells were then further incubated for 24 hr and fixed with 4% paraformaldehyde for 30 min. For analysis of neurite outgrowth, cells (more than 200 /well) were randomly photographed using a KEYENCE microscope (Biozero BZ-9100, Osaka, Japan). Neurite lengths were measured using the BZ-H1C software. All measurements were made in duplicate. Differences between groups were compared by Kolmogorov-Smirnov test, with P < 0.001 considered to denote a significant difference.

### Knockdown experiments

siRNA constructs targeting of the *nur77* gene (sense: UCC AGU GGC UCU GAU UAC UAU GGA A antisense: UUC CAU AGU AAU CAG AGC CAC UGG A)^20^, the *creb* gene (siRNA ID: s135440), the *synapsin* 1 gene (siRNA ID: s128739), and a negative universal control (Cat# 46–537) (ncRNA) were obtained from Life Technologies (Carlsbad, CA). We confirmed that these siRNAs sufficiently reduced target gene expressions using qPCR (see Supplementary Fig. S1 on line).

Cells were seeded onto 6-well plates at 1.0 × 10^5^ cell/cm^2^ and incubated for 16 hr, and then transfected with 100 nM *nur77* siRNA and 5 nM *creb* and *synapsin1* siRNA or with ncRNA using Lipofectamine®RNAiMAX (Life Technologies). After 48 hr, the medium was replaced with 10 µM forskolin in DMEM supplemented with 1% (v/v) FBS, and the cells were incubated for a further 1 hr and 24 hr. Then, the cells were used for RNA extraction or immunoblot analysis.

For measurement of neurite lengths of PC12 cells transfected with siRNAs directed against target mRNAs, the cells were plated onto 24-well plates at 1.5 × 10^4^ cells/cm^2^ and incubated for 16 hr. We then co-transfected siRNAs (100 nM) against target mRNAs with 0.125 nM pCX-EGFP using Lipofectamine®RNAiMAX (Life Technologies). After 48 hr, the cells were treated with 10 µM forskolin in DMEM supplemented with 1% (v/v) FBS and incubated for another 24 hr, and then fixed with 4% (w/v) paraformaldehyde for 30 min. For analysis of neurite outgrowth, GFP-positive cells (more than 200 /well) were randomly photographed and measured 24 hr after initial treatment with forskolin. All measurements were made in duplicate.

### RNA extraction and Quantitative real-time PCR (qPCR)

Total RNA was collected with ISOGEN (Nippongene, Toyama, Japan) and extracted with phenol-chloroform. cDNA was then synthesized using the ReverTraAce® qPCR RT Kit (TOYOBO, Osaka, Japan). 25 ng of cDNA was used as template for StepOnePlus^TM^ real-time PCR system (Applied Biosystems, USA) using THUNDERBIRD® Probe qPCR Mix (TOYOBO, Osaka, Japan).

The data were analyzed with delta delta Ct method and normalized to the amounts of *gapdh* RNA expression in each sample. PCR primers used were those of TaqMan® gene expression assay kits for *nur77* (Assay ID: Rn00666994_g1), *creb* (Assay ID: Rn06140207_g1), *synapsin1* (Assay ID: Rn00569468_m1), *tubulin, beta 3 class III* (Assay ID: Rn01431594_m1), *neurod1* (Assay ID: Rn00824571_s1), and *gapdh* (Assay ID: Rn01775763_g1). All measurements were made in triplicate. Data are presented as the mean ± SEM (n = 3). Statistical significance for each time was determined by Student’s t-test. **p < 0.01.

### Immunoblot analysis

Cells were seeded onto 6 cm dishes at 1.0 × 10^5^ cell/cm^2^. After 16 hr, the medium was replaced with DMEM supplemented with 1% (v/v) FBS, and the cells were then treated with 10 µM forskolin for 0–24 hr, respectively. Immunoblot analysis were performed as described previously^73^. The first antibody (anti-phospho CREB (Ser133) antibody (Merck), anti-CREB antibody (Cell Signaling Technology, Danvers, MA, USA), anti-Nur77 antibody (Abcam, Cambridge, UK), anti-Synapsin1 antibody (Abcam), anti-NeuroD antibody (Cell Signaling Technology), anti-β-tubulin III antibody (Sigma) or anti-GAPDH antibody (Merck)) was loaded onto the membrane after blocking with 5% skimmed-milk (Nakarai, Kyoto, Japan), followed by incubation with a horseradish peroxidase-conjugated secondary antibody (MBL CO., LTD., Nagoya, Japan). The bands were detected by ECL Select Western Blotting Detection System Reagent or ECL Prime Western Blotting Detection System Reagent and visualized with an Image Quant LAS 4000 (all GE Healthcare Life Sciences, Little Chalfont, UK). All measurements were made in triplicate. Quantitative analysis was performed by determining the immunofluorescence intensity of the target protein(s) using ImageJ. Data are presented as the mean ± SEM (n = 3). Statistical significance for each time was determined by Student’s t-test. *p < 0.05.

### Chromatin immunoprecipitation (ChIP) assay

ChIP assays were performed as described previously^20^ with some modifications. The diluted chromatin of 5 × 10^6^ cells was incubated with anti-phospho CREB (Ser133) antibody (Merck), anti-CREB antibody (Cell Signaling Technology) or normal rabbit IgG (Cell Signaling Technology) for 12–16 hr at 4 °C. Immune complexes were bound to Protein G sepharose beads preblocked with salmon sperm DNA and bovine serum albumin for 120 min at 4 °C. The beads were washed once each with low-salt wash buffer (0.1% (w/v) SDS, 1% (v/v) Triton-X100, 2 mM EDTA, 150 mM NaCl, and 20 mM Tris-HCl at pH 8.0), high-salt wash buffer (500 mM NaCl wash buffer), LiCl wash buffer (0.25 M LiCl, 1% NP-40, 1% (v/v) deoxycholate, 1 mM EDTA, and 10 mM Tris-HCl at pH 8.0), and twice with TE buffer (10 mM Tris-HCl at pH 8.0 and 1 mM EDTA). Immune complexes bound to Protein G beads were incubated overnight at 65 °C and treated with 100 µg/ml proteinase K for 1 hr at 56 °C before extraction once with phenol/chloroform. The DNA was then precipitated with ethanol containing glycogen as a carrier and resuspended in 20 µl of dH_2_O. DNA was analyzed by qPCR using following PCR probes (sense: GAT CAA ACA ATC CGC GCT CCC anti-sense: CAC CTC TTA AGC GCT CCG TGA, and TaqMan® MGB probe: TAT GGC CAA AGC TC). The ChIP enriched DNA levels were then normalized to input DNA. All measurements were made in duplicate. Data are presented as the mean ± SEM (n = 3). Statistical significance was determined by Student’s t-test. **p < 0.01, ^##^p < 0.01.

### Luciferase assays

A promoter region of the rat *nur77* gene (WT) was cloned from rat genome DNA using PCR and KOD Plus (TOYOBO) and inserted into a pGL3 basic plasmid vector (Promega, Madison, WI, USA) at Kpn I and Hind III sites. Reporter plasmids constructed of fragments of the rat nur77 promoter region (−242, −222, −78, −49) were obtained using a KOD-Plus Mutagenesis Kit (TOYOBO). Promoter fragments were generated by PCR using the following primers: GCG GGT ACC CAG GGC TTG GGG TAG GGG TGG (WT promoter forward primer), GCG AAG CTT CGG CCG GCT CCC GCT CCC CGT (WT promoter reverse primer), CCG CGC TCC CTG CGT CAA TGG (−242 promoter forward primer), GGT ACC TAT CGA TAG AGA AAT GTT CTG GC (−242 promoter reverse primer), GAA CCC CGC GTG CGT CAC GC (−222 promoter forward primer), GGT ACC TAT CGA TAG AGA AAT GTT CTG GC (−222 promoter reverse primer), CCG GGC CGT GTG CGT CAG TG (−78 promoter forward primer), GGT ACC TAT CGA TAG AGA AAT GTT CTG GC (−78 promoter reverse primer), CCC CTC TCC ATG CGT CAC GGA GC (−49 promoter forward primer) and GGT ACC TAT CGA TAG AGA AAT GTT CTG GC (−49 promoter reverse primer).

Site-directed mutagenesis of CRE sites within the −242, −222 and −78 nur77 promoters was also achieved using the KOD-Plus Mutagenesis Kit (TOYOBO). Promoter fragments containing substituted nucleotide sequences were generated by PCR using the following primers: TGA TGG AAC CCC GCG TGC GT (−242 promoter forward primer), ACG CAG GGA GCG CGG ATT G (−242 promoter reverse primer), TGC GCG CGC AGA CAT TCC AGG C (−222 promoter forward primer), ACG CAC GCG GGG TTC CAT TGA C (−222 promoter reverse primer), TGG TGG CGC CCC CGC CCC TCT C (−78 promoter forward primer), and ACG CAC ACG GCC CGG CGA GC (−78 promoter reverse primer). The nucleotide sequences of all inserts were confirmed using a 3130xl Genetic Analyzer (Thermo Fisher Scientific, MA, USA).

Plasmids with the appropriate *nur77* promoters were transfected with the phRL-TK plasmid (Promega) using Lipofectamine 2000 (Life Technologies). After 48 hr, transfected cells were then established as untreated or stimulated cells for 1 hr with 10 µM forskolin. After stimulation, the cells were lysed and the activities of firefly and Renilla luciferase were measured using the Dual-luciferase reporter assay system (Promega). Firefly luciferase activities were normalized to the Renilla control. All measurements were made in duplicate. Data are presented as the mean ± SEM (n = 3). Statistical significance for each time was determined by Student’s t-test. **p < 0.01, ^##^p < 0.01 and *p < 0.05.

## Supplementary information

Supplementary Figure and Tables.
