# Supplementary material for: Molecular mechanism of nur77 gene expression and downstream target genes in the early stage of forskolin-induced differentiation in PC12 cells
Source: Sci Rep. 2020 Apr 14;10:6325. doi: 10.1038/s41598-020-62968-y (PMC7156746; doi:10.1038/s41598-020-62968-y)
Supplement: Supplementary file 1 — Supplementary Figure and Tables. [file 41598_2020_62968_MOESM1_ESM.pdf]

Appendix

Molecular mechanism of *nur77* gene expression and downstream target genes in the early stage of forskolin-induced differentiation in PC12 cells

Hiroki Maruoka, Ryosuke Yamazoe, Ryota Takahashi, Keisuke Yatsuo, Daiki Ido, Yuki Fuchigami, Fumiya Hoshikawa and Koji Shimoke[a, *]

[a]Laboratory of Neurobiology, Department of Life Science and Biotechnology, Faculty of Chemistry, Materials and Bioengineering, 3-3-35, Yamate-cho, Suita, Osaka 564-8680, Japan

*** To whom correspondence should be addressed:** Laboratory of Neurobiology, Department of Life Science and Biotechnology, Faculty of Chemistry, Materials and Bioengineering, Kansai University, 3-3-35, Yamate-cho, Suita, Osaka 564-8680, Japan Tel.: +81 6 6368 1121 ex.5859, Fax: +81 6 6330 3770.
E-mail address: shimoke@kansai-u.ac.jp (K.Shimoke)

Fig. S1

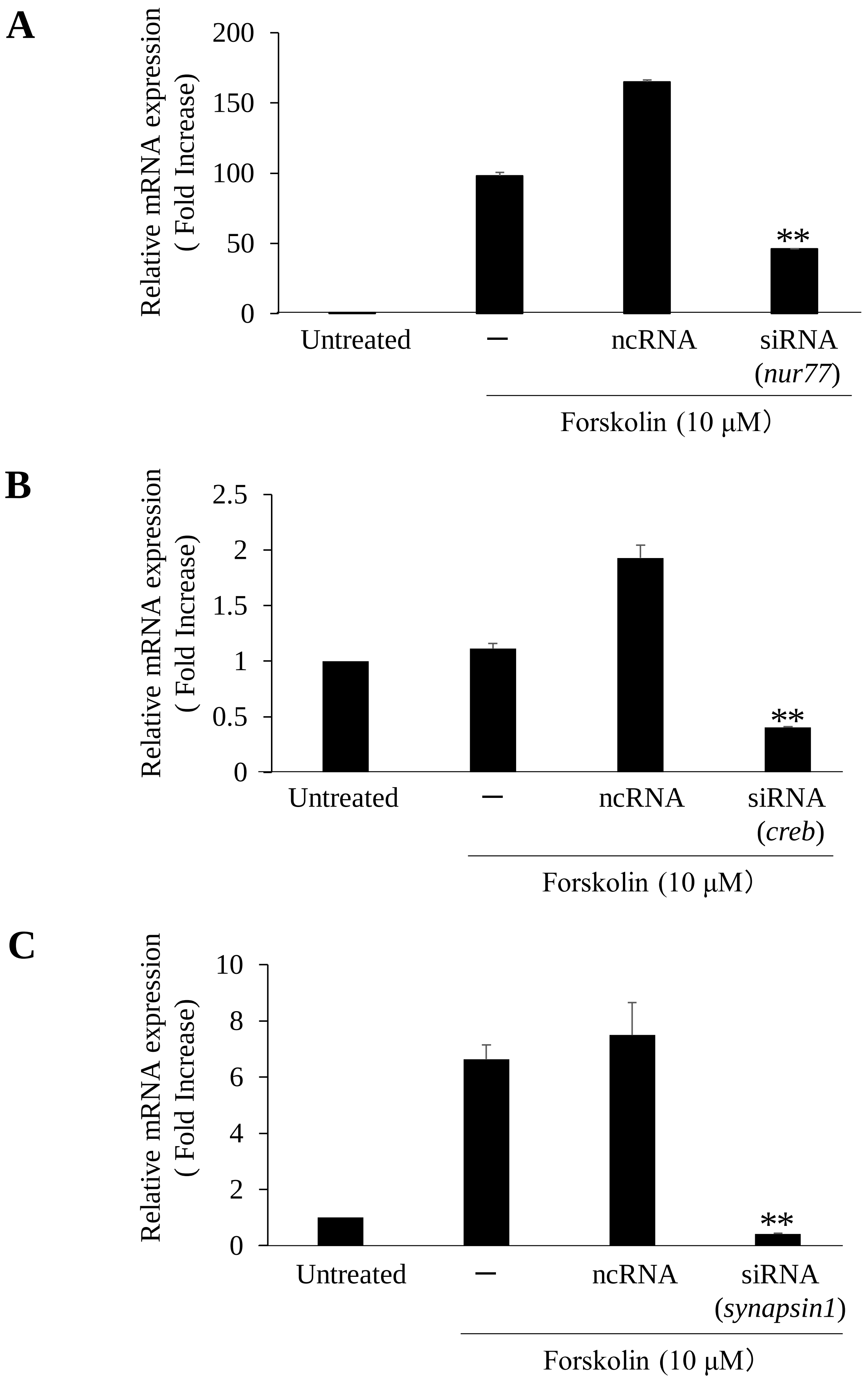

Fig.S1 Quantification of mRNAs in the siRNA-treated cells. PC12 cells transfected with siRNA against *nur77* (A), *creb* (B) and *synapsin1* (C) mRNA or ncRNA (negative universal control siRNA) were treated with or without 10 μM forskolin for 1 hr (*nur77* , *creb*) or 24hr (*synapsin1*). *nur77*, *creb*. *synapsin1* and *gapdh* mRNA were detected by qPCR. *nur77*, *creb* and *synapsin1* mRNA were normalized against *gapdh* mRNA.
**P< 0.01 compared with ncRNA. All measurements were made in duplicate.

Tables

Table S1. Number and percentage of forskolin-induced PC12 cells with more than 20 μm neurite

	untreated	Forskolin
Cells (>20 μm neurite)	1	171
Total cells	209	219
% of cells	0.5	78.1

Table S2. Number and percentage of forskolin-induced nur77 knockdown PC12 cells with more than 20 μm neurite

	untreated	Forskolin	nur77-ncRNA	nur77-siRNA
Cells (>20 μm neurite)	7	145	138	61
Total cells	205	221	215	213
% of cells	3.4	65.6	64.2	28.6

Table S3. Number and percentage of forskolin-induced creb knockdown PC12 cells with more than 20 μm neurite

	untreated	Forskolin	creb-ncRNA	creb-siRNA
Cells (>20 μm neurite)	11	167	151	61
Total cells	214	213	200	237
% of cells	5.1	78.4	75.5	25.7

Table S4. Number and percentage of forskolin-induced synapsin1 knockdown PC12 cells with more than 20 μm neurite

	untreated	Forskolin	synapsin1-ncRNA	synapsin1-siRNA
Cells (>20μm neurite)	5	180	163	3
Total cells	212	201	234	230
% of cells	2.4	89.6	69.7	1.3
